# Supplementary material for: Quercetin Reduces the Development of 2,3,7,8-Tetrachlorodibenzo-p-dioxin-Induced Cleft Palate in Mice by Suppressing CYP1A1 via the Aryl Hydrocarbon Receptor
Source: Nutrients. 2022 Jun 13;14(12):2448. doi: 10.3390/nu14122448 (PMC9229746; doi:10.3390/nu14122448)
Supplement: Supplementary file 1 [file nutrients-14-02448-s001.zip › nutrients-1679736-supplementary.pdf]

Group	1st RLU		average RLU	TCDD activity	sample activity	suppression rate (%)
CTRL	32955	31806	32381			
TCDD	826539	758225	792382	760002		
Q250	51277	50317	50797		18417	
Q50	39019	38852	38936		6555	
Q25	32380	29439	30910		-1471	
TCDD + Q250	54148	51364	52756		20376	97.3%
TCDD + Q50	36667	37249	36958		4578	99.4%
TCDD + Q25	38462	35561	37012		4631	99.4%

Group	2nd RLU		average RLU	TCDD activity	sample activity	suppression rate (%)
CTRL	24551	26105	25328			
TCDD	764166	773503	768835	743507		
Q250	35194	36560	35877		10549	
Q50	29947	29635	29791		4463	
Q25	25015	25968	25492		164	
TCDD + Q250	36206	38724	37465		12137	98.4%
TCDD + Q50	30583	28514	29549		4221	99.4%
TCDD + Q25	25841	28980	27411		2083	99.7%

Group	3rd RLU		average RLU	TCDD activity	sample activity	suppression rate (%)
CTRL	43650	53367	48509			
TCDD	1153688	1220048	1186868	1138360		
Q250	62880	56349	59615		11106	
Q50	37218	42409	39814		-8695	
Q25	38430	35221	36826		-11683	
TCDD + Q250	59226	65455	62341		13832	98.8%
TCDD + Q50	35112	41453	38283		-10226	100.9%
TCDD + Q25	33043	42826	37935		-10574	100.9%

A formula: TCDD activity = TCDD's RLU - CTRL's RLU

sample activity = sample's RLU - CTRL's RLU

suppression rate (%) = TCDD activity - sample activity / TCDD activity × 100

Figure S1
